# Salidroside can target both P4HB-mediated inflammation and melanogenesis of the skin

**DOI:** 10.7150/thno.47413

**Published:** 2020-08-13

**Authors:** Xiu-Juan Ding, Zhi-Yuan Zhang, Jing Jin, Jing-Xia Han, Yan Wang, Kai Yang, Yu-Yan Yang, Hong-Qi Wang, Xin-Tong Dai, Cheng Yao, Tao Sun, Cai-Bin Zhu, Hui-Juan Liu

**Affiliations:** 1 Cheermore Cosmetic Dermatology Laboratory, Shanghai, China.; 2 Tianjin Key Laboratory of Early Druggability Evaluation of Innovative Drugs, Tianjin International Joint Academy of Biomedicine, China.; 3 State Key Laboratory of Medicinal Chemical Biology and College of Pharmacy, Nankai University, Tianjin, China.; 4 Department of Gastroenterology and Hepatology, General Hospital, Tianjin Medical University, Tianjin Institute of Digestive Disease, Tianjin, China.; 5 Quality Management Department, Shijiazhuang Food and Drug Inspection Center, Hebei, China.

**Keywords:** Salidroside, tyrosinase, prolyl 4-hydroxylase beta polypeptide (P4HB), interferon regulatory factor 1 (IRF1), upstream stimulatory factor 1 (USF1)

## Abstract

**Rationale:** Many external factors can induce the melanogenesis and inflammation of the skin. Salidroside (SAL) is the main active ingredient of *Rhodiola*, which is a perennial grass plant of the Family Crassulaceae. This study evaluated the effect and molecular mechanism of SAL on skin inflammation and melanin production. It then explored the molecular mechanism of melanin production under ultraviolet (UV) and inflammatory stimulation.

**Methods:** VISIA skin analysis imaging system and DermaLab instruments were used to detect the melanin reduction and skin brightness improvement rate of the volunteers. UV-treated Kunming mice were used to detect the effect of SAL on skin inflammation and melanin production. Molecular docking and Biacore were used to verify the target of SAL. Immunofluorescence, luciferase reporter assay, CO-IP, pull-down, Western blot, proximity ligation assay (PLA), and qPCR were used to investigate the molecular mechanism by which SAL regulates skin inflammation and melanin production.

**Results:** SAL can inhibit the inflammation and melanin production of the volunteers. SAL also exerted a protective effect on the UV-treated Kunming mice. SAL can inhibit the tyrosinase (TYR) activity and TYR mRNA expression in A375 cells. SAL can also regulate the ubiquitination degradation of interferon regulatory factor 1 (IRF1) by targeting prolyl 4-hydroxylase beta polypeptide (P4HB) to mediate inflammation and melanin production. This study also revealed that IRF1 and upstream stimulatory factor 1 (USF1) can form a transcription complex to regulate TYR mRNA expression. IRF1 also mediated inflammatory reaction and TYR expression under UV- and lipopolysaccharide-induced conditions. Moreover, SAL derivative SAL-plus (1-(3,5-dihydroxyphenyl) ethyl-β-d-glucoside) showed better effect on inflammation and melanin production than SAL.

**Conclusion:** SAL can inhibit the inflammation and melanogenesis of the skin by targeting P4HB and regulating the formation of the IRF1/USF1 transcription complex. In addition, SAL-plus may be a new melanin production and inflammatory inhibitor.

## Introduction

Many external factors, such as solar ultraviolet (SUV) radiation and inflammatory stimuli, can cause melanin production 1. Among these factors, UV radiation is the main pathogenic factor of melanogenesis 2. Pathologic melanogenesis can cause skin inflammation and produce pro-inflammatory cytokines, which may accelerate skin aging 3. Intense and intermittent sun exposure boosts the normal function of melanocytes 4, and cutaneous melanoma is associated with intermittent and constant sun exposure. Melanin synthesis inhibitors are widely used in medicine and cosmetics. Melanin synthesis inhibitors, including kojic acid and its derivatives (*e.g.*, kojic acid ether derivatives), are tyrosinase (TYR) inhibitors. However, these agents only target the downstream melanogenesis pathway. In addition, these agents have serious side effects, such as weak carcinogenicity 5 and discoloration 6, which may be due to the irritability pathological compensatory function of cells. Finding new and safe melanogenesis inhibitors has application potential in medicine and cosmetics.

The pathway of melanin biosynthesis is formed by a series of enzymatic and non-enzymatic reactions. TYR is a copper-glycoenzyme involved in the biosynthesis of widespread melanin pigments. TYR contains two active sites (CuA and CuB) 7. TYR is a rate-limiting enzyme for melanin synthesis. Its abnormal overexpression is responsible for the pathologic melanogenesis of humans 8. Upstream stimulatory factor 1 (USF1) is a transcriptional regulator of TYR 9. The molecular mechanism by which IRF1 regulates TYR expression has not been elucidated.

*Rhodiola* is a perennial grass plant of the Family Crassulaceae. It usually lives in harsh alpine areas (*e.g.*, Tibet) and has a variety of pharmacological activities. It is also a traditional medicinal material commonly used in Asia. *Rhodiola* has anti-fatigue, anti-aging, immune regulation, anti-fibrosis, anti-tumor 10, and free radical-scavenging 11 activities. *Rhodiola* can also protect keratinocytes from UV radiation 12 and inhibit melanin production. The hydroalcoholic extract of *Rhodiola* and its hydrolysate can inhibit melanin production by regulating the cAMP response element-binding protein/microphthalmia-associated transcription factor (MITF)/TYR pathway 13. Salidroside (SAL) is the main active ingredient of *Rhodiola* 14, 15. The addition of SAL can substantially inhibit TYR activity and melanin production in B16F10 melanoma cells 16. However, the mechanism by which SAL inhibits melanin production has not been fully elucidated.

In this work, we evaluated the effect of SAL on inflammation and melanogenesis. SAL exerts anti-inflammatory effect and inhibits melanin production in A375 cells. Thus, we further studied its target and molecular mechanism. Results showed that SAL can inhibit the activity of TYR and regulate the ubiquitination degradation of IRF1 by targeting prolyl 4-hydroxylase beta polypeptide (P4HB) to regulate inflammation and TYR mRNA expression. IRF1 and USF1 could form a transcription complex to regulate TYR mRNA expression. Moreover, a series of SAL derivatives was synthesized to find additional effective active molecules, and results showed that the derivative 1-(3,5-dihydroxyphenyl) ethyl-β-D-glucoside (SAL-plus) demonstrated better effect than SAL.

## Methods

### Materials

SAL was purchased from Beka Chemical Technology Co., Ltd. (Shanghai, China). The antibodies to P4HB and USF1 were purchased from Abcam (Cambridge, MA, USA). The antibodies to IRF1 were obtained from Cell Signaling Technology (Danvers, MA, USA). The antibodies to TYR were purchased from Affinity (Cincinnati, USA). P4HB overexpression plasmid, P4HB siRNA, and USF1 siRNA were purchased from GenePharma (Shanghai, China). IRF1 siRNA and overexpression plasmid were purchased from Sino Biological Inc. (Beijing, China). Cell PHD assay kit was purchased from GENMED (Shanghai, China). Melanin-stimulating hormone (α-MSH) and MG132 were purchased from Med Chem Express (New Jersey, USA). Dulbecco's modified eagle medium (DMEM) and fetal bovine serum (FBS) were purchased from Hyclone (Logan, Utah, USA). Trypsin lyophilized powder was purchased from Sangon Biotech (Shanghai, China).

### Cell culture

A human melanogenic cell line (A375) and a human immortal keratinocyte line (HaCaT) were obtained from KeyGen Biotech (Nanjing, China) in 2013 and authenticated by short tandem repeat genotyping. Mycoplasma was analyzed using a Mycoplasma quantitative polymerase chain reaction (qPCR) detection kit (Sigma) before experiment. Cells were grown in DMEM supplemented with 10% FBS and maintained at 37 °C in a humidified atmosphere containing 5% CO_2_.

### TYR activity assay in A375 cells

The inhibitory effect of SAL on TYR activity was measured using a spectrophotometric method. Cells were grown in a 24-well plate and treated with different concentrations of SAL or SAL-plus for 72 h. The cells were harvested by trypsinization and washed three times with ice-cold phosphate-buffered saline (PBS). Lysis buffer was prepared by combining 0.1 M sodium phosphate buffer (pH 6.8) containing 0.1% Triton X-100 and protease inhibitors. The cells were disrupted by sonication at 4 °C, and the lysate was centrifuged at 16,000 rpm for 30 min. Protein concentration was quantified using a bicinchoninic acid assay kit, and then the cell lysates were adjusted to the same protein concentration using the lysis buffer. The reaction system, including cellular extracts, 2 mg/mL levodopa (L-Dopa), and 0.1 M sodium phosphate buffer (pH 6.8), was incubated in a 96-well plate at 37 °C for 2 h. Absorbance was measured at 450 nm on a microplate reader.

### Melanin content assay

Melanin content was detected in accordance with the procedure described by Hosoi *et al.* 17. The cells were seeded in a 6-well plate at 7.5 × 10^5^ cells/well. After the cells adhered, 1 µL of 10 µM α-MSH was added to each well for 24 h, and then the cells were treated with various concentrations of SAL or SAL-plus for 48 h. The harvested cells were washed twice with PBS, resuspended in 1 M NaOH containing 10% dimethyl sulfoxide, and then heated at 80 °C for 1 h. The absorbance of the extracted melanin was read at 405 nm on a microplate reader (Thermo Scientific Varioskan).

### UVB-induced hyperpigmentation in Kunming mice

Kunming mice (female, 6-8 weeks) were maintained in a specific pathogen-free animal care facility. The mice were allowed to acclimatize for 7 days before the experiment. All animal studies were carried out in accordance with the Animal Use Guidelines of National Institutes of Health and the current Chinese Regulations and Standards for the Use of Laboratory Animals. All animal procedures were approved in accordance with the guidelines of the Animal Ethics Committee of Tianjin International Joint Academy of Biotechnology and Medicine. The mice were randomly divided into four groups (n = 6): control, model (treated with the UVB), SAL (UVB-exposed skin was given 0.5% SAL every day for 4 weeks), and SAL-plus (UVB-exposed skin was given 0.5% SAL-plus every day for 4 weeks). Except the control group, the other groups were anesthetized and separate areas (1.5 cm × 1.5 cm) of the back of each animal were exposed to UVB radiation (Waldmann UV800, Herbert Waldmann GmbH, Philips TL/12 lamp emitting 280-305 nm). The total UVB dose was 500 mJ/cm^2^ per exposure. The animals were exposed to UVB radiation three times a week for 2 consecutive weeks, and the mice continued to be treated with SAL or SAL-plus for 2 weeks. All mice were euthanized after 2 weeks of treatment. Skin tissues were collected and fixed with 4% paraformaldehyde at 4 °C for 24 h. The tissues were dehydrated and embedded in paraffin in accordance with standard procedures. Serial sections of 4 μm thickness were obtained. Fontana-Masson staining and hematoxylin-eosin (HE) staining were performed to evaluate the melanin content and pathological changes of the skin tissues.

### Volunteer recruitment

Seventy healthy volunteers aged 20-30 years (24.5 ± 3.3 years) participated in these experiments. All of the volunteers are Asians of similar skin color. All the reagents used in this experiment can be found in the “list of used cosmetics raw materials” (2015, China), which are filed by the China Food and Drug Administration, and we strictly abide by the concentration limit of raw materials. All volunteers were informed and signed the informed consent. All experimental studies were carried out in accordance with the guiding principles for claims and evaluation of cosmetic efficacy (National Institute for food and drug control). The study protocol was approved by the ethics committee of Cheermore Cosmetic Dermatology Laboratory. In the first set of experiment, 30 volunteers were divided into two groups (n = 15): control (treated with basic cream) and SAL (treated with cream containing *Rhodiola* extract containing 0.25% SAL). The reagents were smeared on the face of the volunteers once a day. The VISIA skin analysis imaging system (Canfield scientific, USA) was used to test the effect of SAL on the speckle, red zone, brown spots, UV spots, and violet spots of the skin. In the second set of experiment, 40 volunteers were divided into four groups (n = 10): control (treated with basic cream), *Rhodiola* extract (treated with cream containing *Rhodiola* extract containing 0.25% SAL), vitamin C (VC) group (treated with cream containing 0.2% VC), and kojic acid group (treated with cream containing 0.2% kojic acid). Then, corresponding reagents were smeared on the inside of the arm once a day. DermaLab instruments (Cortex Technology, Denmark) was used to detect melanin reduction, skin brightness improvement, and changes in collagen distribution every week for 4 weeks.

### Western blot analysis

After treatment with SAL, proteins were extracted from A375 cells and analyzed by Western blot. The culture medium was aspirated, each dish was washed with PBS, and the lysis buffer containing protease inhibitors was added to obtain the cell lysate. Protein concentration was determined using the BCA method, and the proteins were separated by sodium dodecyl sulfate-polyacrylamide gel electrophoresis (SDS-PAGE). Then, the proteins were transferred to a polyvinylidene fluoride (PVDF) membrane activated by methanol. After blocking with 5% skim milk powder, the membranes were incubated with primary antibodies against P4HB (Abcam, 1:1000), IRF1 (CST, 1:1000), TYR (Affinity, 1:1000 dilution), USF1 (Abcam, 1:1000), ubiquitin (Ub, Affinity, 1:1000), histone H3 (Affinity, 1:1000), or glyceraldehyde 3-phosphate dehydrogenase (GAPDH, Affinity, 1:1000). The samples were incubated with primary antibody overnight at 4 °C. After washing out the primary antibody, the PVDF membrane was further incubated with horseradish peroxidase-labeled secondary antibodies (Affinity, 1:5000). Finally, the target proteins were visualized using enhanced chemiluminescence substrate reagents (Millipore, USA).

### Immunofluorescence (IF) staining

A375 cells overexpressed with IRF1 or treated with SAL were plated on cell climbing films in 24-well plates for 48 h. The cells were fixed in 3.7% paraformaldehyde for 15 min, treated with 0.1% Triton X-100 for 10 min, and then incubated with 3% bovine serum albumin for 30 min. Then, the cells were incubated with the primary antibodies, anti-IRF1 (1:50 dilution) and anti-USF1 (1:50 dilution) antibodies, and then incubated with the corresponding fluorescent secondary antibodies. Finally, the nuclei were made visible by staining with 4'6-diaminido-2-phenylindole diluted by 1:10,000 at room temperature for 5 min. The proteins were visualized by confocal microscopy (Nikon, Japan).

### Proximity ligation assay (PLA)

The interaction between IRF1 and USF1 proteins was detected by performing a similar double immunostaining protocol with the secondary antibodies replaced by PLA probes obtained from the Duolink kit (Sigma Aldrich). Hybridization between two PLA plus and minus probes produced a fluorescent signal only when the distance between the two proteins was ≤ 40 nm.

### Protein ubiquitination assay

A375 cells were divided into four groups: siControl, siP4HB, control (treated with vehicle), and SAL (treated with 50 µM SAL). The cells were treated in different conditions for 24 h. Then, the cells were lysed using immunoprecipitation (IP) lysis buffer (Thermo Fisher Scientific, Inc.), and the total protein in the lysate served as the “input” sample. Protein A/G beads were incubated with IRF1 antibody or IgG (negative control) for 6-8 h to form the immune complex. The immune complex was incubated with the cell lysate overnight, and then the immune complex on the beads was eluted by the elution buffer. Subsequently, the “input” and “elute” samples were tested by Western blot analysis using antibodies against Ub, IRF1, or P4HB.

### Co-IP assay

The collected A375 cells were rinsed with cold PBS and lysed in IP lysis buffer, and the total protein in the lysate served as the “Input” sample. Then, protein A/G beads were incubated with anti-IRF1 antibody and anti-USF1 antibody or IgG (negative control) separately for 6-8 h to form the immune complex. The beads were incubated with cell lysate overnight and eluted using the elution buffer, which was considered as the “Elute.” Subsequently, the “Input” and “Elute” samples were detected by Western blot using antibodies against IRF1 or USF1.

### RNA isolation and qPCR

Total RNA was extracted from the cells using RNeasy Mini Kit (Qiagen, China) and treated with RNase-free DNase I (Qiagen, China) to remove residual genomic DNA. Each 25 µL of the reaction system contained 1.5 µg of total RNA, and cDNA was synthesized using the High-Capacity cDNA Kit (Applied Biosystems). qPCR reactions were performed on Applied Biosystems ViiA 7 using gene-specific primers and iTaq SYBR green (Bio-Rad Laboratories). Relative gene expression was calculated using the standard curve method with GAPDH as an internal control. The primer sequences used are shown in Table S1. The experimental result obtained by qPCR is the Ct value. Three independent repeated tests were performed. ∆Ct = Ct (experimental group)-Ct (GAPDH). Then, the 2^^-ΔCt^ values were calculated separately. The 2^^-ΔCt^ value of the samples divided by the average value of 2^^-∆Ct^ in the control is the 2^^-ΔΔCt^ value. Compared with the control group, if the 2^^-ΔΔCt^ value of the experimental group decreases, it means that the expression of the detected gene (TYR) decreases; by contrast, it means that the expression of the detected gene (TYR) increases.

### Whole-genome gene expression chip analysis

The cells were treated with or without SAL for 48 h and then lysed to isolate RNA, and gene expression was detected with whole-genome gene expression chip (Genergy). The differentially expressed genes (|logFC| > 1.5) were analyzed by Gene Ontology (GO) analysis to detect changes in molecular functions, biological processes, and cellular components induced by SAL. The STRING database (https://string-db.org) was used to analyze the protein-protein interactions.

### Luciferase reporter assay

Luciferase reporter assay was performed to detect the transcriptional activation effect of IRF1 on TYR mRNA expression in A375 cells. We cloned TYR-binding sites of the promoter (TBSs-pro) into the pGL3 luciferase reporter plasmid. The A375 cells were transfected with the IRF1 overexpression plasmid. After 48 h of transfection, luciferase activities were detected using a Dual-Luciferase Reporter Gene Assay Kit (Promega).

### Statistical analysis

The final results were expressed as mean **±** standard deviation (SD). Data were statistically analyzed using Student's t test. Statistical significance was considered at *p* < 0.05.

## Results

### Effect of *Rhodiola* extract on the skin of volunteers

**Figure 1:**
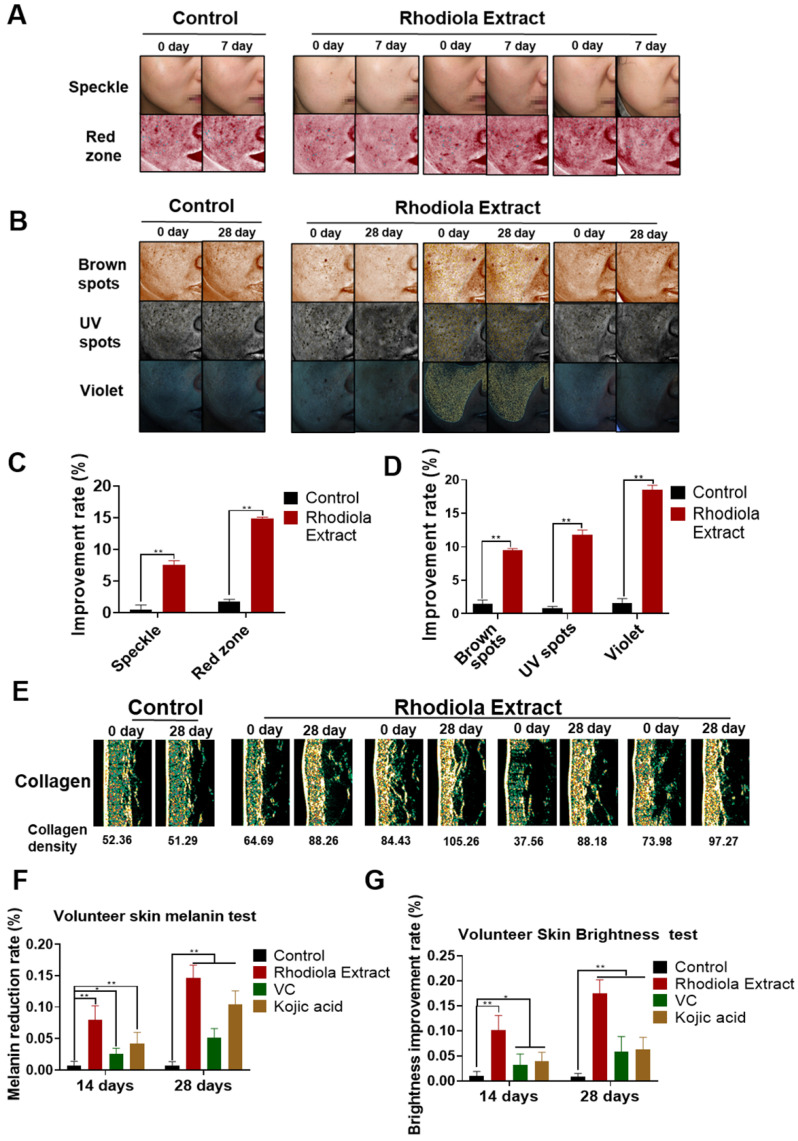
** Effect of *Rhodiola* extract on the skin of volunteers. A, B.** Skin image detected by VISIA imaging system showing the speckle, red zone, UV spots, violet spots, and brown spots of volunteers. **C, D.** Statistical analyses of the improvement rate of speckle, red zone, UV spots, violet spots, and brown spots. **E.** Collagen content in the arm detected by the Dermalab instrument with skin test probes. **F, G.** Melanin reduction and brightness improvement rate detected by Dermalab. Data are expressed as mean ± SD (^*^P < 0.05, ^**^P < 0.01).

Considering that SAL is not in the “list of used cosmetics raw materials” (2015, China), we used *Rhodiola* extract with a high concentration of SAL to detect the effect of SAL on the skin of the volunteers. The VISIA imaging system was used to analyze the effect of *Rhodiola* extract on the volunteers. Results showed that the speckle, red zone, UV spots, brown spots, and violet spots improved in the volunteers treated with *Rhodiola* extract (Figure 1A-B). The percentage of speckle and red zone in the *Rhodiola* extract-treated group remarkably improved in the first week (Figure 1C), indicating that SAL has obvious anti-inflammatory effects. The UV spots, violet spots, and brown spots on the skin of the *Rhodiola* extract-treated volunteers substantially improved in the fourth week (Figure 1D), indicating that SAL can reduce skin pigmentation.

Dermalab instrument with skin test probes was used to detect the skin characteristics of the arm, such as collagen density, melanin content, and skin brightness, on days 0, 7, 14, and 28 to evaluate the whitening effect of SAL and compare the effect of SAL with those of VC and kojic acid. Results showed that the collagen content in the *Rhodiola* extract-treated group was higher than that in the control group (Figure 1E). The melanin reduction rate and skin brightness improvement rate of the *Rhodiola* extract-treated group were higher than those of the VC- and kojic acid-treated groups. The melanin reduction rate of the *Rhodiola* extract-treated group was 7.98% in the second week and 14.67% in the fourth week. The improvement rate of skin brightness in the *Rhodiola* extract-treated group was 10.12% in the second week and 17.51% in the fourth week (Figure 1F-G). These results suggest that SAL can inhibit melanin production.

### Inhibitory effect of SAL on melanin biosynthesis in A375 cells

**Figure 2:**
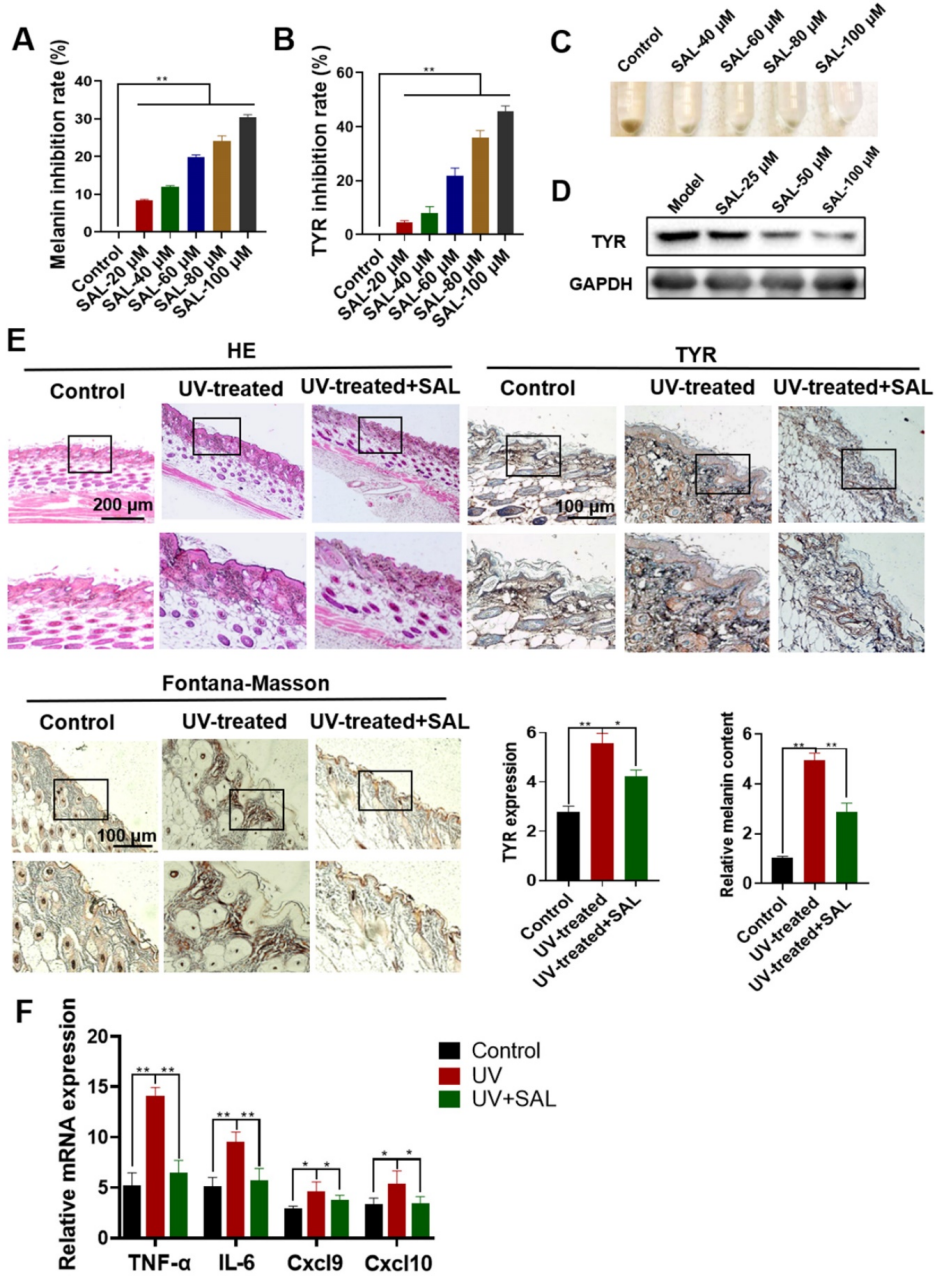
** Inhibitory effect of SAL on melanin biosynthesis in A375 cells. A.** Inhibition rate of melanin production in A375 cells treated with different concentrations of SAL. **B.** Inhibition rate of the TYR activity of A375 cells treated with SAL. **C.** Changes in the melanin content of A375 cells treated with SAL. **D.** Western blot analysis of TYR expression in A375 cells treated with SAL. **E.** Effect of SAL on the skin of UV-treated mice as detected by HE staining of paraffin sections, IHC staining, and Fontana-Masson staining. **F.** Expression of TNF-α-, IL-6-, Cxcl9-, and Cxcl10-related mRNAs in the skin of SAL-treated UV irradiation mice. Data are expressed as mean ± SD (^*^P < 0.05, ^**^P < 0.01).

The effect of SAL on melanin synthesis was detected in A375 cells. Results showed that SAL can inhibit melanin production dose dependently (Figure 2A). SAL also inhibited the activity of TYR in a dose-dependent manner (Figure 2B). The color of the cell precipitates treated with SAL also indicated that SAL inhibited pigment synthesis in A375 cells (Figure 2C). Western blot experiment showed that SAL inhibited TYR expression dose dependently (Figure 2D).

### SAL can inhibit UV-induced melanin production and inflammation in mice

HE staining was used to detect the protective effect of SAL on the skin of the UV-treated mice. Compared with the control group, the model group had a thicker epidermal layer, a disordered tissue structure, local proliferation, and inflammatory cell infiltration (Figure 2E). SAL treatment inhibited the UV-induced skin structure disorder and reduced the epidermal layer thickness. IHC results showed that the expression of TYR protein increased in the UV-treated group (*P*<0.01). TYR expression reduced in the SAL-treated group. Fontana-Masson staining results showed that the melanin expression in the model group substantially increased, whereas that in the SAL-treated group reduced. We examined the expression of mRNAs related to the TNF-α, IL-6, Cxcl9, and Cxcl10 in the skin tissues of the three groups to detect the anti-inflammatory effect of SAL. Results showed that the mRNA contents of the inflammatory-related factors in the UV-treated group were remarkably higher than those in the control group, whereas those in the SAL-treated group were reduced. These results indicate that SAL has a remarkable inhibitory effect on inflammatory-related factors (Figure 2F).

### SAL can target TYR and P4HB

**Figure 3:**
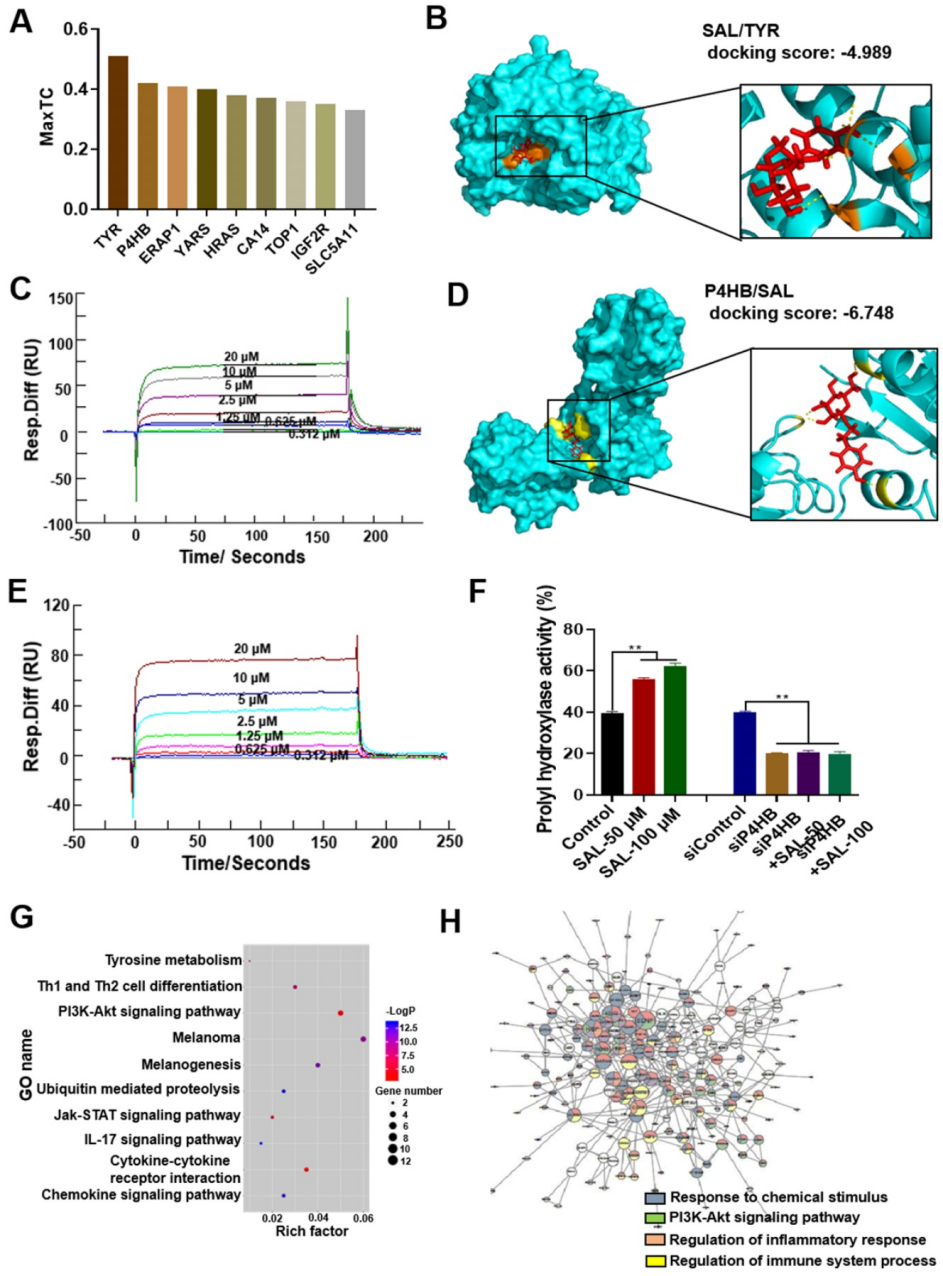
** SAL can bind to TYR and P4HB. A.** Prediction of the target of SAL through SEA search server. **B.** SAL exhibited good binding activity to TYR as determined by molecular docking. **C.** SAL exhibited good binding activity to TYR as determined by Biacore assay. **D.** SAL exhibited good binding activity to P4HB as determined by molecular docking. **E.** SAL exhibited good binding activity to P4HB as determined by Biacore assay. **F.** Effect of SAL on the prolyl hydroxylase activity in A375 cells or P4HB knockdown cells. **G, H.** Effect of SAL on the proteomics of A375 cells (GO analysis) and protein-protein interaction network. Data are expressed as mean ± SD (^*^P < 0.05, ^**^P < 0.01).

The target of SAL was predicted through the similarity ensemble approach search server (http://sea.bkslab.org/), and the results are shown in Figure 3A. The MaxTC values of P4HB and TYR were prominent. Thus, we speculated that SAL plays a pharmacological role by targeting TYR and P4HB. Molecular docking was used to verify the hypothesis. The docking score was used to evaluate the binding ability between the ligand and the receptor. The absolute value of the docking score represents the strength of the binding ability. The docking score of SAL and TYR was -4.989, and the docking score of SAL and P4HB was -6.748 (Figure 3B and 3D). Subsequently, surface plasmon resonance assay (Biacore 3000) was used to verify the interaction between the proteins and SAL. Results showed that SAL can bind with TYR (K_D_ = 9.48 × 10^-6^ M, Figure 3C) and P4HB (K_D_ = 1.38 × 10^-5^ M, Figure 3E). Further experiments verified that SAL could target and affect prolyl hydroxylase activity (Figure 3F). SAL can increase prolyl hydroxylase activity in a dose-dependent manner. However, SAL exerted no remarkable effect on the prolyl hydroxylase activity in P4HB knockdown cells using siRNA (siP4HB). These results verified the interaction between SAL and P4HB.

The gene expression profile changes between control and SAL groups were analyzed. The differentially expressed genes were analyzed by GO analysis. The enrichment function is shown in Figure 3G. The rich factor showed the ratio of the genes participating in the GO term to the total number of genes, which reflected the enrichment degree of the GO term. The size of the scatters shows the number of genes, and the color of the scatters shows the *P* value. Results indicated that SAL mainly influenced the melanin production pathway, the ubiquitination pathway, and the inflammation-related pathways. The protein-to-protein interaction network was used to analyze the enriched genes. Results showed that these genes were enriched to regulate the cellular response to chemical stimulus, the regulation of inflammatory response, and so on (Figure 3H). These results further verified the effect of SAL on melanogenesis and inflammation.

### P4HB regulates the expression of TYR and the ubiquitination degradation of IRF1

**Figure 4:**
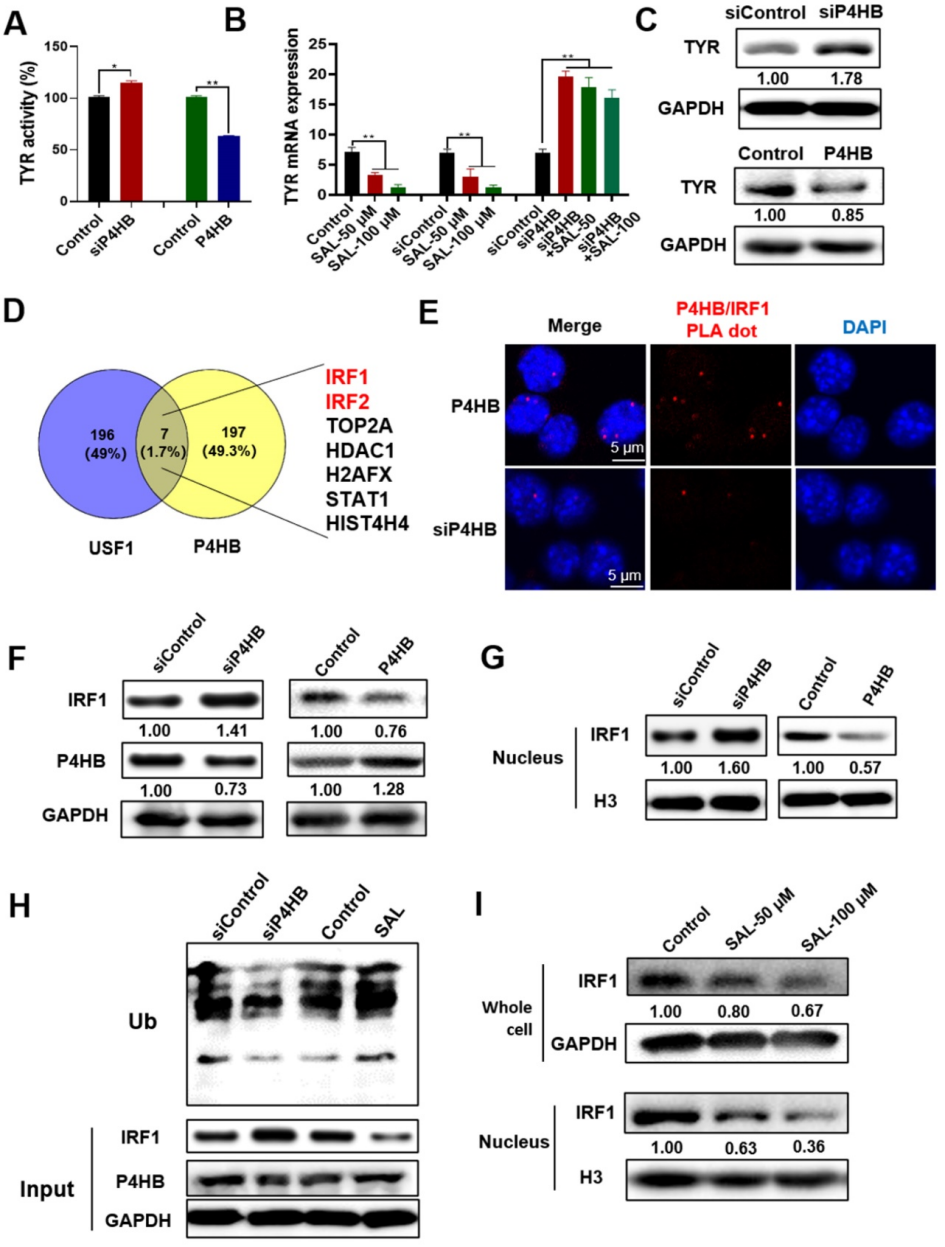
** P4HB regulates the ubiquitination degradation of IRF1. A.** Changes in TYR activity in P4HB overexpression or knockdown A375 cells. **B.** Changes in TYR mRNA expression in Sal treated, P4HB overexpression, and P4HB knockdown A375 cells. **C.** Western blot analysis of TYR expression in P4HB overexpression or knockdown A375 cells. **D.** Proteins have interactions with P4HB and USF1 analyzed by FpClass. **E.** Interaction of P4HB and IRF1 in A375 cells detected by PLA. **F.** Western blot analysis of the expression of IRF1 and P4HB in P4HB overexpression or knockdown A375 cells. **G.** Western blot analysis of IRF1 expression in the nucleus of A375 cells after P4HB overexpression or knockdown. **H.** Effect of SAL on the ubiquitination of IRF1 as determined by Western blot. **I.** Western blot analysis of IRF1 expression in whole cells and nucleus of SAL-treated A375 cells. Data are expressed as mean ± SD (^*^P < 0.05, ^**^P < 0.01).

In this work, we found that SAL could inhibit the activity and expression of TYR (Figure 2D). The mechanism of SAL on TYR expression was explored. Considering that SAL can target P4HB, we first evaluated the effect of P4HB on TYR production. We examined the changes in TYR activity after the knockdown or overexpression of P4HB in A375 cells. TYR activity increased in P4HB knockdown cells using the siRNA of P4HB. TYR activity decreased in P4HB overexpression cells (Figure 4A). TYR mRNA expression remarkably increased in P4HB knockdown A375 cells (Figure 4B). Although SAL could inhibit the mRNA expression of TYR dose dependently, SAL slightly affected the expression of TYR mRNA in the cells after the P4HB gene interference, indicating that SAL may inhibit the TYR mRNA expression by targeting P4HB. The expression of TYR protein increased in P4HB gene knockdown cells and decreased in P4HB overexpression cells (Figure 4C). These results showed that P4HB can influence the expression of TYR. Therefore, we further researched the mechanism by which P4HB regulates TYR expression. USF1 can bind to the TYR promoter and activate the transcription of the *TYR* gene. We analyzed the proteins having physical interaction with P4HB and USF1 by using the FpClass database (http://dcv.uhnres.utoronto.ca/FPCLASS/ppis/) and employed the Venn method to analyze the proteins that interact with P4HB and USF1. P4HB and USF1 had no physical interaction, and the Venn intersection showed that the IRFs can interact with P4HB and USF1 and may mediate the effect of P4HB on TYR expression (Figure 4D). Considering that P4HB is a key enzyme in protein ubiquitin-proteasome degradation, we speculated that SAL can promote the ubiquitination and degradation of IRF1 through targeting P4HB. Thus, SAL may inhibit the formation of the IRF1/USF1 transcription complex, inhibit the expression of the *TYR* gene, and ultimately inhibit the production of melanin.

The interaction between P4HB and IRF1 in the nucleus was observed by PLA assay. Results showed that P4HB interacts with IRF1 (the red dot indicates the interaction of the protein), and the interaction in the P4HB interference group was considerably weaker than that in the control group (Figure 4E). P4HB overexpression cells and knockdown cells were used to detect the effect of P4HB on IRF1 to further evaluate the interaction between P4HB and IRF1 (Figure 4F). Results showed that IRF1 expression decreased remarkably in P4HB overexpression cells and increased in P4HB knockdown cells. IRF1 is a transcriptional regulatory factor. Thus, we further detected its expression in the nucleus. The expression of IRF1 also decreased in P4HB overexpression cells and increased in P4HB knockdown cells (Figure 4G). These results showed that P4HB can interact with IRF1 and affect its expression. The IP method was used, and the ubiquitination level of IRF1 was detected to investigate whether or not P4HB can regulate IRF1 ubiquitination. The IP product was used to detect the content of ubiquitinated IRF1, and results showed that the content of ubiquitinated IRF1 can be reduced by P4HB knockdown and can be increased by SAL treatment (Figure 4H). The IRF1 content in the cells and nucleus affected by SAL was also detected. Compared with the control group, the SAL-treated group had a remarkably lower IRF1 content in the cytoplasm and the nucleus (Figure 4I). These results indicated that P4HB can influence the ubiquitination of IRF1, and SAL can promote the ubiquitination of IRF1 by targeting P4HB.

### IRF1/USF1 forms a transcription complex to promote melanin production

**Figure 5:**
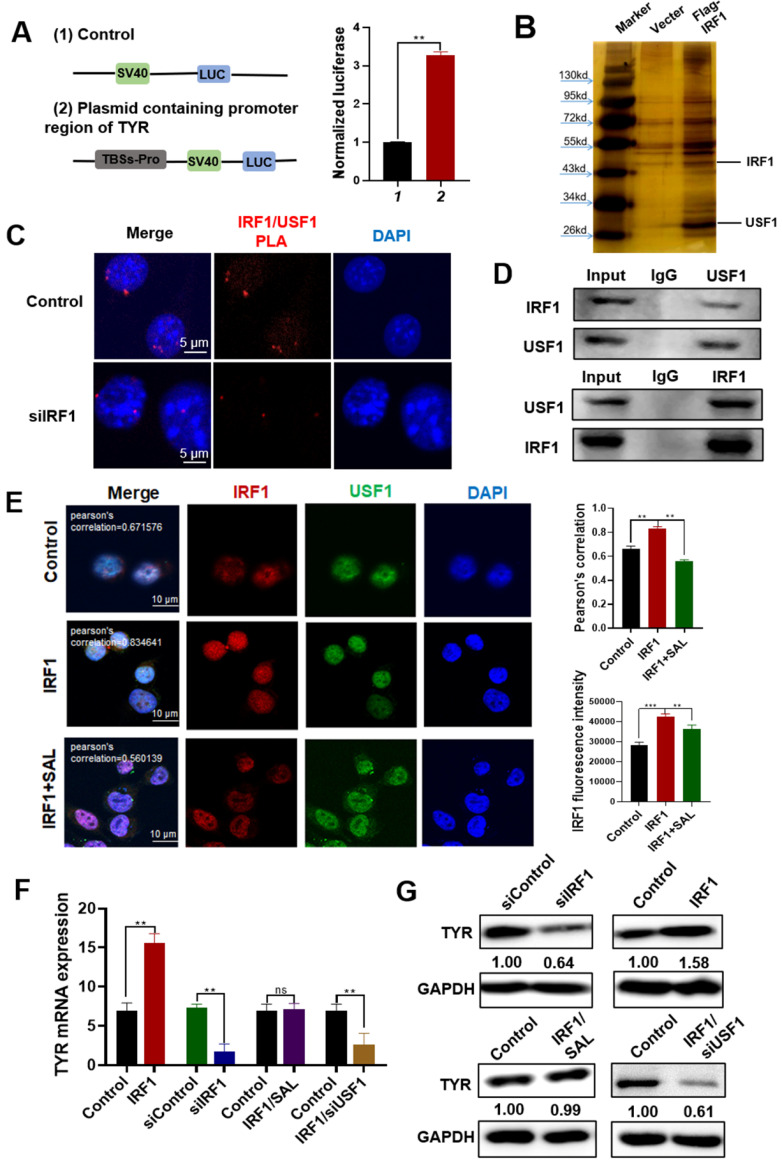
** IRF1 and USF1 form a transcription complex to promote melanin production in A375 cells. A.** Luciferase reporter assay of the transcriptional activation effect of IRF1 on TYR in A375 cells. **B.** Pull-down analysis of the proteins that interact with IRF1. Cellular extracts from Flag-IRF1 overexpression A375 cells were subjected to affinity purification with anti-Flag affinity columns and eluted with Flag peptide. Elutes were separated by SDS-PAGE and stained with silver. **C.** PLA analysis of the interaction between IRF1 and USF1 in A375 cells. **D.** Co-IP analysis of the interaction of IRF1 and USF1 in A375 cells. **E.** Interaction of IRF1 and USF1 verified by IF experiment. **F, G.** Changes in TYR mRNA and protein expression in IRF1 overexpression and IRF1 knockdown cells. Data are expressed as mean ± SD (^*^P < 0.05, ^**^P < 0.01).

We further verified the effect of IRF1 on TYR expression and its interaction with USF1. The TYR-binding sites on the promoter (TBSs-pro) were cloned into the pGL3 luciferase reporter to confirm the effect of IRF1 on TYR transcription. Results showed that IRF1 overexpression could enhance the activity of the firefly luciferase reporter gene, which indicated that IRF1 can bind to the promoter of TYR and increased the transcription of TYR (Figure 5A). We performed pull-down experiments to verify the complex of IRF1 and USF1. The experiment showed that IRF1 co-purified with USF1 protein (Figure 5B). The co-localization of IRF1 and USF1 proteins in the nucleus was determined using PLA, Co-IP, and IF assays. The PLA results showed that IRF1 can interact with USF1, which can be reflected by the red dot of the probe. The interaction between IRF1 and USF1 remarkably reduced after IRF1 interference (Figure 5C). Co-IP analysis further confirmed that IRF1 interacted with USF1 protein (Figure 5D). The results of IF measurement showed that after IRF1 overexpression, the colocalization of IRF1 and USF1 significantly enhanced, but the colocalization in the IRF1 overexpression plus SAL-treated group reduced (Figure 5E). These results indicated that IRF1 can interact with USF1 to form a transcription complex. SAL can reduce IRF1 expression and reduce its interaction with USF1.

The mRNA expression of TYR in IRF1 overexpression or knockdown cells was detected by qPCR (Figure 5F). Results showed that the expression of TYR mRNA increased after IRF1 overexpression, and its expression was reduced after IRF1 interference. The mRNA expression of TYR did not change remarkably in the IRF1 overexpression combined with SAL-treated group. In the cells of overexpression IRF1 combined with USF1 knockdown (siUSF1), TYR expression also decreased, which indicated that the effect of IRF1 on TYR expression was mediated by USF1. We further examined the expression of TYR in A375 cells by Western blot (Figure 5G). Results showed that the expression of TYR increased in IRF1 overexpression cells, and the expression of TYR decreased in IRF1 knockdown cells. TYR expression did not change remarkably in the IRF1 overexpression combined with SAL-treated group. TYR expression also decreased in the IRF1 overexpression and USF1 knockdown group. These results showed that IRF1 can form a transcription complex with USF1 and regulate the expression level of TYR. SAL can decrease the IRF1 content in the A375 cells to inhibit the formation of the IRF1/USF1 transcription complex.

### SAL inhibits LPS/UV-induced melanin production and inflammation

**Figure 6:**
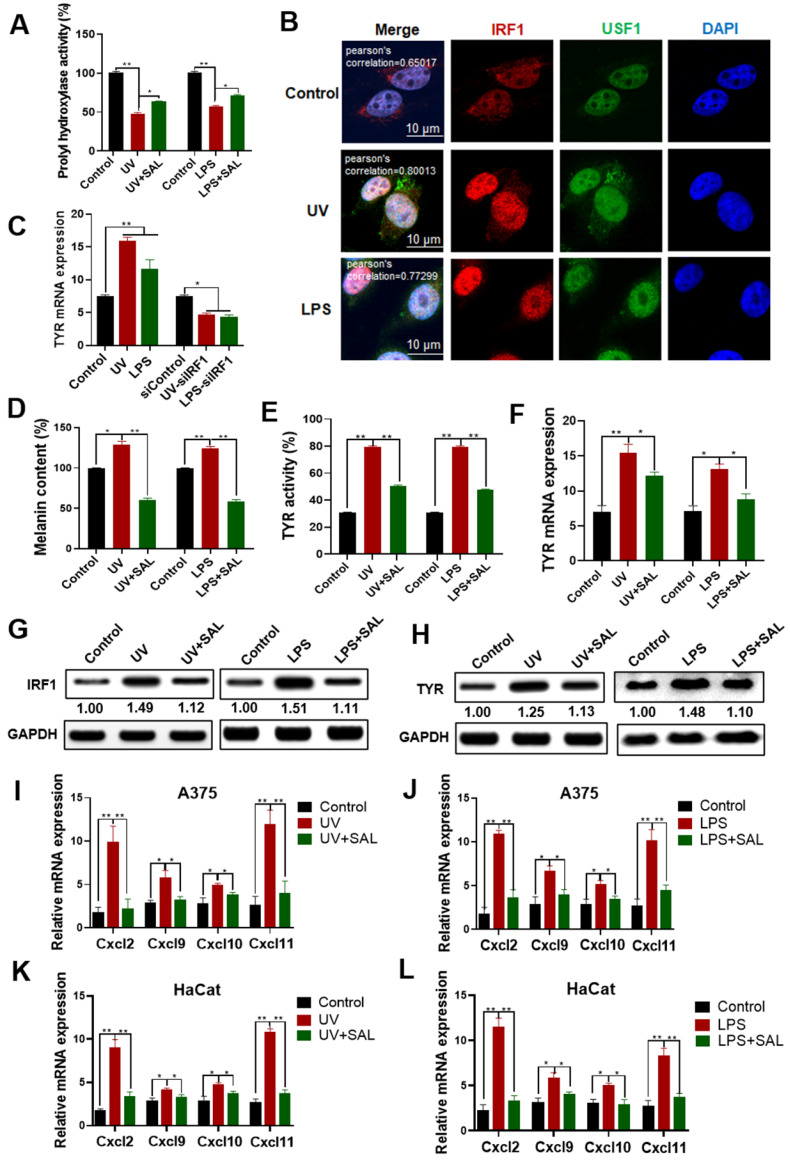
** SAL inhibits LPS/UV-induced inflammation and melanin production in A375 cells. A.** Effect of SAL on the prolyl hydroxylase activity in UV- and LPS-treated A375 cells. **B.** Interaction of IRF1 and USF1 in A375 cells induced by UV irradiation and LPS detected by immunofluorescence. **C.** Effect of SAL on TYR mRNA expression in UV- and LPS-treated A375 cells. **D-F.** Effect of SAL on the melanin content, TYR activity, and TYR mRNA expression of UV- or LPS-treated A375 cells. **G, H.** Effect of SAL on the expression of IRF1 and TYR proteins in UV- and LPS-treated A375 cells as detected by Western blot. **I, J.** Effects of SAL on the mRNA expression levels of Cxcl2, Cxcl9, Cxcl10, and Cxcl11 in UV- and LPS-treated A375 cells. **K, L.** Effect of SAL on the mRNA expression levels of Cxcl2, Cxcl9, Cxcl10, and Cxcl11 in UV- and LPS-treated HaCaT cells. Data are expressed as mean ± SD (^*^P < 0.05, ^**^P < 0.01).

We tested the activity of prolyl hydroxylase in A375 cells after UV induction or lipopolysaccharide (LPS) induction. Results showed that the activity of prolyl hydroxylase remarkably reduced after UV and LPS induction (Figure 6A). The activity of prolyl hydroxylase was inhibited in the SAL-treated group compared with the UV-treated and LPS-treated groups. Results of IF assay (Figure 6B) showed that the co-localization of IRF1 and USF1 proteins in the UV-induced and LPS-induced groups was stronger than that in the control group. These results indicated that UV and LPS can stimulate the formation of the IRF1/USF1 transcription complex.

Results of TYR mRNA expression in the UV- and LPS-treated A375 cells showed that UV and LPS could induce the expression of TYR (Figure 6C). However, the TYR mRNA expression showed no obvious change in the IRF1 interference cells after UV and LPS treatment compared with the control group. These results showed that IRF1 mediated the UV- and LPS-induced melanin synthesis. The melanin contents and TYR activity in the UV- and LPS-induced groups were remarkably higher than those in the control group (Figure 6D-E). The melanin contents and TYR activity in the UV- and LPS-induced groups reduced after SAL treatment.

The expression of TYR mRNA in the UV- and LPS-induced groups was higher than that in the control group, whereas the expression of TYR mRNA was lower in the SAL-treated groups than in the UV- and LPS-treated groups (Figure 6F). Furthermore, we detected the expression of IRF1 in the cells induced by UV and LPS. Results showed that IRF1 expression increased in the cells treated with UV and LPS, and SAL could reduce IRF1 expression under UV and LPS conditions (Figure 6G). TYR expression was detected by Western blot, and results showed that UV and LPS increased TYR expression and SAL inhibited TYR expression under UV and LPS conditions (Figure 6H).

The effect of SAL on the mRNA expression of intracellular inflammatory-related factors, including Cxcl2, Cxcl9, Cxcl10, and Cxcl11, was detected in the UV- and LPS-treated cells. Results showed that the mRNA levels of Cxcl2, Cxcl9, Cxcl10, and Cxcl11 in the UV- and LPS-treated A375 cells increased compared with those in the control group, and SAL could reduce these mRNA expression levels (Figure 6I-J). The mRNA expression levels of Cxcl2, Cxcl9, Cxcl10, and Cxcl11 also increased in the HaCaT cells in the UV- and LPS-treated groups, and SAL inhibited the mRNA expression of inflammatory factors in the HaCaT cells (Figure 6K-6L). These results indicated that IRF1 mediated the effect of UV and LPS in the A375 cells and SAL can inhibit the LPS/UV-induced melanin production and inflammation.

### Synthesis and activity evaluation of SAL derivatives

**Figure 7:**
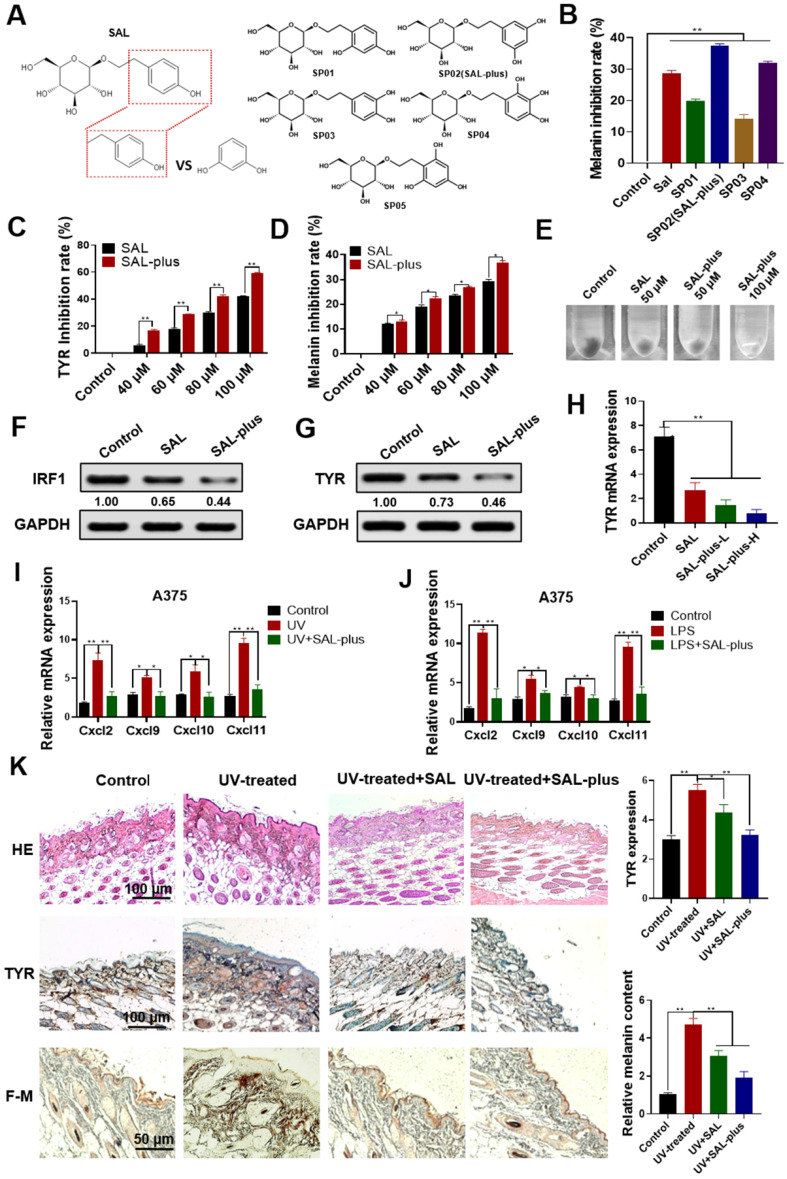
** Synthesis and activity evaluation of SAL derivatives. A.** Structural formula of synthetic SAL derivatives (SAL-plus). **B.** Inhibitory effect of SAL-plus on melanin production in A375 cells. **C-E.** Comparative detection of the TYR activity inhibition rate and melanin inhibition rate of SAL and SAL-plus in A375 cells. **F, G.** Western blot analysis of IRF1 and TYR protein expression changes in SAL- and SAL-plus-treated A375 cells. H. Analysis of the mRNA expression levels of TYR in SAL and SAL-plus (L: 50 µM, H: 100 µM) treated A375 cells. **I, J.** mRNA expression levels of related inflammatory factors Cxcl2, Cxcl9, Cxcl10, and Cxcl11 in UV, LPS, and SAL-plus treated A375 cells. **K.** Effect of SAL-plus on the skin of UV-treated mice as detected by HE staining, IHC staining, and Fontana-Masson staining of paraffin sections. Data are expressed as mean ± SD (^*^P < 0.05, ^**^P < 0.01).

Resorcinol is a strong TYR inhibitor but causes great skin irritation and side effects. Therefore, we considered the chemical modification of SAL to make its side chain form a resorcinol group to enhance the melanin inhibition ability of SAL. The para-substituted substituents of compounds containing resorcinol structure can improve the TYR inhibitory activity of compounds 10. We synthesized SAL derivatives and detected the activity of these derivatives. The chemical structures of the SAL derivatives are shown in Figure 7A. The melanin inhibition rate of the synthesized SAL derivative was tested. Results showed that SP02 (SAL-plus) showed the best inhibitory effect on melanin production among the derivatives (Figure 7B). The chemical synthesis methods and characterization of SAL-plus are shown in the supplementary materials (Figure S1-3). The effects of SAL and SAL-plus on melanin content at different concentrations and TYR activity were evaluated (Figure 7C-E). Results showed that SAL-plus could inhibit melanin content and TYR activity in a dose-dependent manner and showed better effect than SAL. The inhibitory effect of SAL-plus on the expression of IRF1 and TYR was also better than that of SAL (Figure 7F-G). SAL-plus also inhibited the mRNA expression of TYR (Figure 7H) and the mRNA expression of inflammatory factors induced by UV and LPS in A375 cells (Figure 7I- J).

The effect of SAL-plus on UV-induced melanin production in mice was also detected (Figure 7K). HE staining was used to detect the protective effect of SAL-plus on the skin. The model group had a thickened epidermal layer, disordered tissue structure, inflammatory cell infiltration, and local proliferation compared with the control group. SAL-plus can improve the skin structure induced by UV, and the epidermal layer treated by SAL-plus was thinner than that in the UV group. IHC results showed that the expression of TYR protein in the UV-treated group increased (*P* < 0.01), whereas that in the SAL-plus treated group decreased. Fontana-Masson staining results showed that the melanin expression increased in the model group and reduced in the SAL-plus treated group. These results showed that SAL-plus could inhibit the UV-induced melanin production *in vivo*, and its effect was better than that of SAL. We also evaluated the safety of SAL-plus. The skin of the mice was continuously smeared with SAL-plus for 4 weeks, and SAL-plus (2.5%-5%) had no remarkable adverse effect on the skin of the mice compared with the control group. The results are shown in supplementary materials (Figure S4-5).

## Discussion

The results of the present study indicated that SAL can inhibit the inflammation and melanin production of the volunteers, and SAL also exerted protective effects on the UV treated Kunming mice. SAL can inhibit the activity of TYR, the expression of TYR, and the expression inflammation factors in A375 cells. Target prediction showed that SAL can bind to P4HB protein. We further investigated the mechanism by which P4HB regulates melanin production to explain the mechanism of SAL on melanin production, especially under UV and inflammatory conditions.

The transcriptional regulation of TYR protein is currently under research, and the basic helix-loop-helix factor (essential for melanocyte differentiation) encoded by the ophthalmopathy gene can pass through the M-box near the promoter and the E-box activates the TYR promoter 18, 19. MITF is a cell type-specific factor that can specifically activate the *TYR* gene's promoter activity through the CATGTG motif of the TYR distal element (TDE), which activates the transcription of the *TYR* gene. USF also contains a basic helix-loop-helix structure and a leucine zipper structure; thus, USF1 can also bind to TDE to activate the transcription of the *TYR* gene 20. Galibert *et al.* studied the ability of pigment cells to respond to UV radiation as part of UV-induced tanning reactions and showed that the transcription factor USF1 and microphthalmia-related transcription factors jointly bind the conserved E-box element in the TYR promoter and regulate the expression of TYR 9. This study investigated whether or not the transcriptional regulation of USF1 requires the participation of other proteins, especially under UV- and inflammation-induced conditions. The results of the present work showed that USF1 can form a transcription complex with IRF1 to regulate the transcription of the *TYR* gene. The regulatory effect of IRF1 on melanin production and TYR protein expression has not been reported in previous works. This work showed that IRFI can form a transcription complex with USF1 to regulate the expression of TYR protein and then regulate melanin production.

The mechanisms underlying inflammation and melanin formation remain unclear at present. Exposure to acute SUV leads to IFNγ upregulation and downstream pSTAT1/IRF-1/uSTAT1 signaling in the epidermis and increases the proinflammatory chemokine mRNAs in the epidermis 21, including Cxcl9, Cxcl10, and Ccl2. In addition, keratinocytes can change the melanin production of melanocytes by producing soluble factors under inflammatory conditions 22. IRFs are a family of transcription factors playing important roles in inflammatory regulation, cytokine signaling, cell death, and cell differentiation 23, 24. This work showed that IRF1 can mediate inflammation and melanogenesis under UV and LPS stimulation. IRF1 also participated in UV- and LPS-induced melanin production. IRF1 accumulated in cells under UV and LPS conditions, and IRF1 can form a transcription complex with USF1 to promote the transcription of *TYR*, increase the expression of TYR proteins, and thereby promote melanin synthesis. SAL can also inhibit the mRNA expression of the above proinflammatory chemokines by reducing the content of IRF1 in A375 and HaCaT cells under UV and LPS conditions.

IRF1 is primarily degraded through the ubiquitination-dependent degradation pathway. In ubiquitination, the protein needs to be hydroxylated under the action of prolyl hydroxylase, recognized by E3 ubiquitin ligase, modified by ubiquitination, and then degraded by proteasome. P4HB is a prolyl hydroxylase subunit that forms an α2β2 tetramer with the 59-kDa α subunit (P4Hα) to play a role in regulating prolyl hydroxylation 25. P4Hα contains a peptide-substrate binding domain and an enzyme active site. The 55-kDa β-subunit P4HB independently functions as a protein disulfide isomerase (PDI). As a P4H subunit, PDI retains the enzyme in the inner cavity of the endoplasmic reticulum through its C-terminal KDEL retention signal, which maintains the α-subunit in a soluble and active form and promotes its refolding. This work showed that P4HB can influence the ubiquitination degradation of IRF1. SAL may promote the formation of α2β2 tetramer by regulating the activity of P4HB, enhance the activity of prolyl hydroxylase, and promote the ubiquitination degradation of IRF1. Furthermore, SAL inhibited the IRF1/USF1 transcription complex formation; thus, SAL inhibited the expression of TYR and inhibited melanin production.

Intense and intermittent sun exposure boosts the normal function of melanocytes, resulting in their proliferation and increasing melanin production. Cutaneous melanoma is related to intermittent and constant sun exposure 4, 19, 26. This stimulation may occur because of increased synthesis of melanocyte stimulating hormone receptors, which is concurrent with the repair of DNA damage caused by sun radiation 27. Thus, SAL may exert preventive effects on skin injury and tumorigenesis caused by ultraviolet, excessive sunlight, and inflammation.

Resorcinol is a strong TYR inhibitor but may cause great skin irritation and side effects. The para-substituted substituents of compounds containing resorcinol structure are beneficial to improve the TYR inhibitory activity of these compounds 10. Therefore, we designed and synthesized SAL derivatives with resorcinol. These derivatives can improve the inhibitory effect of SAL on melanin production. We found that SAL-plus exerted better effects than SAL and demonstrated no obvious side effects; thus, SAL-plus is expected to be used as an inhibitor of melanin production.

In summary, this study showed that SAL can inhibit the inflammation and melanogenesis of the skin through targeting P4HB and regulating the formation of the IRF1/USF1 transcription complex. A SAL derivative (SAL-plus) with better ability to inhibit melanin production and inflammation was also synthesized. SAL-plus may be a melanogenesis and inflammation inhibitor in the future.

## Supplementary Material

Supplementary figures and tables.
