# Supplementary material for: Salidroside can target both P4HB-mediated inflammation and melanogenesis of the skin
Source: Theranostics. 2020 Aug 13;10(24):11110–26. doi: 10.7150/thno.47413 (PMC7532676; doi:10.7150/thno.47413)
Supplement: Supplementary file 1 — Supplementary figures and tables. [file thnov10p11110s1.pdf]

Supplementary materials for

Salidroside can target both P4HB-mediated inflammation and melanogenesis of the skin

Xiu-Juan Ding[*,1,3], Zhi-Yuan Zhang[*,1], Jing Jin[*,1], Jing-Xia Han[*,2], Yan Wang[5], Kai Yang[1], Yu-Yan Yang[2,3], Hong-Qi Wang[2,3], Xin-Tong Dai[2,3], Cheng Yao[#,1], Tao Sun[#,2,3,4], Cai-Bin Zhu[#,1], Hui-Juan Liu[#,2,3]

[1]Cheermore Cosmetic Dermatology Laboratory，Shanghai, China.

[2]Tianjin Key Laboratory of Early Druggability Evaluation of Innovative Drugs, Tianjin International Joint Academy of Biomedicine, China

[3]State Key Laboratory of Medicinal Chemical Biology and College of Pharmacy, Nankai University, Tianjin, China.

[4]Department of Gastroenterology and Hepatology, General Hospital, Tianjin Medical University, Tianjin Institute of Digestive Disease, Tianjin, China.

[5]Quality Management Department, Shijiazhuang Food and Drug Inspection Center, Hebei, China.

*These authors have contributed equally to this work.

[#]Correspondence and requests for materials should be addressed to Hui-Juan Liu (liuhuijuanxyz@163.com), Tao Sun (tao.sun@nankai.edu.cn), or Cai-Bin Zhu (alex@cheermorecos.com).

Figures S1-S5

1. Synthesis method of Sal-plus

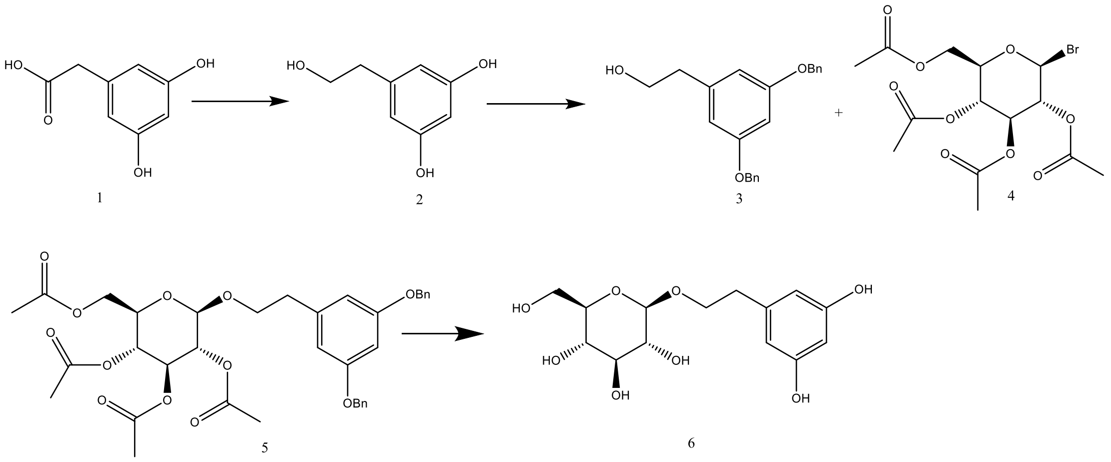

Figure S1. Synthesis route of Sal-plus.

1). Synthesis of compound 2：1.18 g (7.0 mmol) of 3,4-dihydroxyphenylacetic acid (compound 1) and 8.4 ml of BH3/THF (1.0 M in THF) were dissolved in 20 mL of THF and reacted at room temperature for 4 hours. After the reaction, the solvent was removed by rotary evaporation to obtain a crude product (compound 2). Column chromatography purification was used to obtain 1.05g product (compound 2), yield 97%.

2). Synthesis of compound 3：Add 16.9 g (0.05 mol) of compound 2, 6.9 mL (0.06 mol) of (chloromethyl)benzene and 200 mL of acetone to the reaction flask in turn, stir to dissolve, add 8.3 g (0.06 mol) of potassium carbonate and 0.8 g (0.005 mol) of potassium iodide, reflux for 18 h (TLC monitoring). The solvent was distilled off, the residue was poured into 200 mL of water, hydrochloric acid was added dropwise to adjust to pH=7, and the product was extracted with ethyl acetate. Then the organic phase was distilled off by vacuum distillation. Finally, the product (compound 3) was recrystallized from absolute ethanol, which is white solid (210.7 g), yield 88.6%.

3). Synthesis of compound 5: Dissolve 27.3 g compound 3 (0.03 mol) in 200 mL of CH2Cl2, add 14.8 g compound 4 (0.036mol), 2 g of molecular sieve and 55 g (0.018mol) of dry silver carbonate under stirring, and react at room temperature in the dark for 1 day. After filtration, the filter product was washed with an appropriate amount of CH_2Cl_2, and the filtrate was concentrated to obtain compound 5.

4). Synthesis of compound 6 (Sal-plus): Dissolve the above compound 5 in 150 mL of anhydrous methanol, add sodium methoxide 1.6 g (0.03 mol), stir at room temperature for 6 h, adjust to pH=6 with acetic acid, filter, and concentrate the filtrate under reduced pressure to obtain viscous

material. 150 ml of methanol, 0.16 g of palladium carbon was added to the viscous substance and the reaction was stirred for 4 h under hydrogen atmosphere. The final product 5.6 g Sal-plus was obtained by column chromatography purification.

^{1}H-NMR (CD3OD, 400 MHz) δ: 7.06 (2H, d, J = 8.4 Hz), 6.69 (2H, d, J = 8.4 Hz), 4.29 (1H, d, J = 8.0 Hz), 4.02 (1H, m), 3.86 (1H, dd, J = 1.6, 12.4 Hz), 3.68 (2H, overlapped), 3.23-3.37 (3H, overlapped), 3.18(1H, t, J = 8.8 Hz), 2.83 (2H, t, J = 8.0 Hz); (Figure S2)

^{13}C-NMR (CD3OD, 100 MHz) δ: 155.4, 129.6, 129.4, 114.7, 103.0, 76.7, 76.6, 73.7, 70.7, 70.3, 61.4, 35.0. (Figure S3)

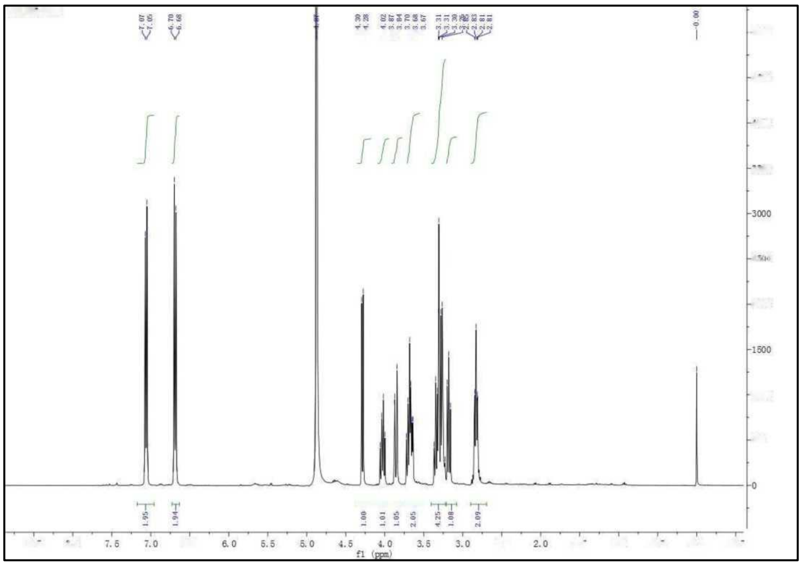

Figure S2. H1-NMR of the Sal-plus.

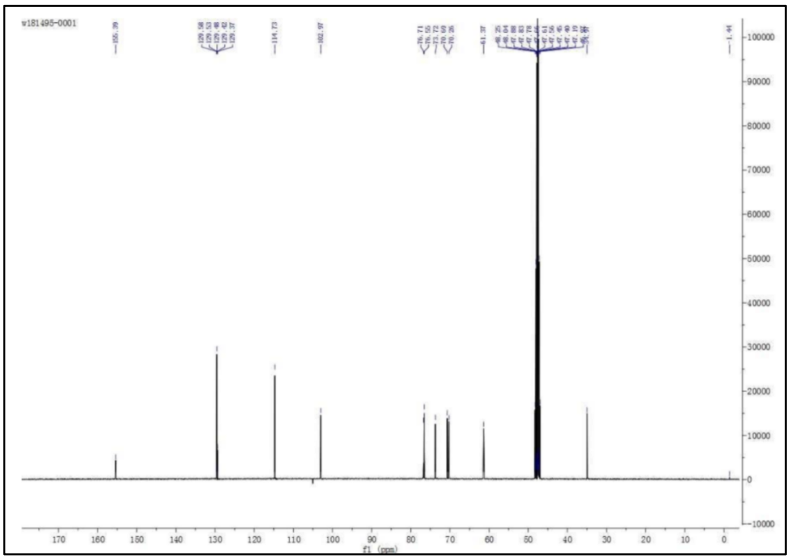

Figure S3. ^{13}C-NMR of the Sal-plus.

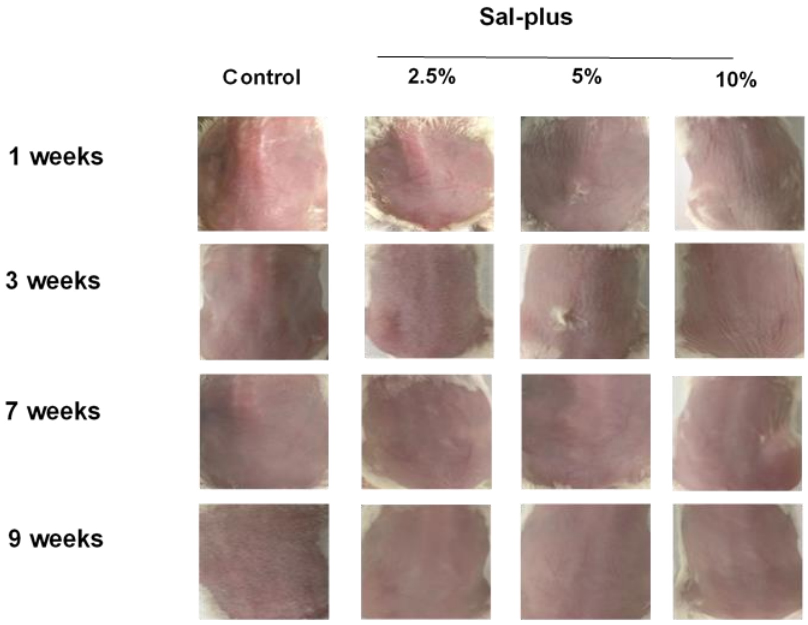

Figure S4. Long-term toxicity test of the Sal-plus (General observation of skin morphology).

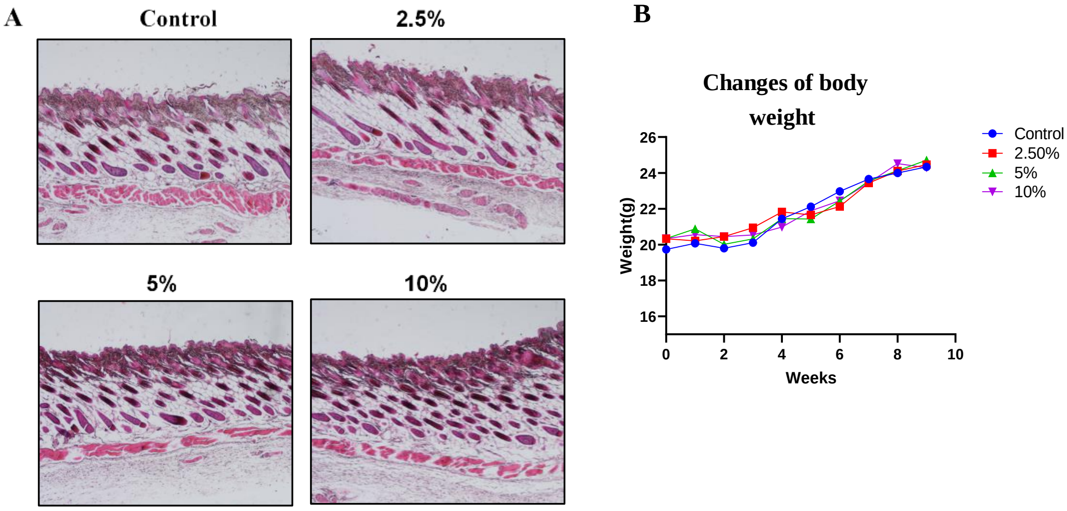

Figure S5. Long-term toxicity test of the Sal-plus (HE staining) and the changes of the body weight.

Table S1. Primers used in qPCR assay.

Gene	Primer
GAPDH forward	GGAGCGAGATCCCTCCAAAAT
GAPDH reverse	GGCTGTTGTCATACTTCTCATGG
TYR forward	GACATAACCGGGAATCCTACA
TYR reverse	CCAAGGAGCCATGACCAG
